# What Can Eye Movements Tell Us about Subtle Cognitive Processing Differences in Autism?

**DOI:** 10.3390/vision3020022

**Published:** 2019-05-24

**Authors:** Philippa L Howard, Li Zhang, Valerie Benson

**Affiliations:** 1 Department of Psychology, Bournemouth University, Bournemouth BH12 5BB, UK; 2 Academy of Psychology and Behaviour, Tianjin Normal University, Tianjin 300074, China; 3 School of Psychology, University of Central Lancashire, Preston PR1 2HE, UK

**Keywords:** autism, eye movements, cognitive processing, social and everyday communication

## Abstract

Autism spectrum disorder (ASD) is neurodevelopmental condition principally characterised by impairments in social interaction and communication, and repetitive behaviours and interests. This article reviews the eye movement studies designed to investigate the underlying sampling or processing differences that might account for the principal characteristics of autism. Following a brief summary of a previous review chapter by one of the authors of the current paper, a detailed review of eye movement studies investigating various aspects of processing in autism over the last decade will be presented. The literature will be organised into sections covering different cognitive components, including language and social communication and interaction studies. The aim of the review will be to show how eye movement studies provide a very useful on-line processing measure, allowing us to account for observed differences in behavioural data (accuracy and reaction times). The subtle processing differences that eye movement data reveal in both language and social processing have the potential to impact in the everyday communication domain in autism.

## 1. What Can Eye Movements Tell Us about Subtle Cognitive Processing Differences in Autism?

Eye tracking is widely used to examine information processing [1] since it is well established that eye movement patterns provide detailed insight into on-going cognitive processing [2]. Autism spectrum disorder (ASD) is a heterogeneous developmental condition characterised by difficulties engaging in everyday social interaction/communication, restricted and repetitive patterns of behaviour, and sensory processing sensitivities [3]. It is widely accepted within the field that these behavioural symptoms are underpinned by information processing differences [4]. Therefore, eye tracking provides an opportunity to examine the nature of on-going cognitive processing in ASD, and to evaluate how any cognitive processing differences might underpin behavioural symptoms in this population. 

In 2009 the research into autism that had utilised eye tracking was reviewed and at this point there were approximately 60 articles published in the field [5]. The chapter reviewed how eye-tracking had been used to explore low-level eye-movement characteristics, perception of complex stimuli, and processing of and attention to social information. The review concluded that basic oculomotor control such as smooth pursuit and saccadic programming appeared to be intact in ASD. However, subtle differences in attention allocation were thought to be present for tasks that required higher-level cognitive and social processing. For example, consistent observations reported enhanced local processing during visual search and atypical allocation of attention for social scenes. The heterogeneity of the disorder, changes across development and the effect of general ability and linguistic level were all shown to impact upon findings from studies reviewed in that chapter. For example, visual sampling or scanning in autism was shown to be affected by the complexity of stimuli social content, task complexity, and symptom profile, including age, symptom severity, the presence of language delay, and social competence. It was proposed that future research ought to take account of specific sub groups of the ASD population, and must also employ more naturalistic stimuli and settings, such as the presentation of dynamic information and investigation of processing in one to one social interactions. 

Since the review was published, there has been a surge in experiments that have used eye tracking to study ASD. A search in Web of Science for “autism” AND ”eye tracking” indicates more than 600 research papers have been published on this topic in the last decade. Many of the suggestions for future research, addressed in the previous review, have been taken on board in these new studies. However, many of the issues raised in relation to inconsistent findings reported in the previous review, are also apparent in the studies reported in the current review. 

The current article reviews some of this more recent literature, with a focus upon what has been learnt about the nature of language and social processing in ASD over the past 10 years. The aim of the review will be to evaluate the contribution of the research to the understanding as to how cognitive processing differences could relate to behavioural symptoms in day-to-day communication in ASD. 

Key findings will be presented at the end of each section, and these will outline observed processing differences in ASD for different paradigms or behavioural comparisons. A summary of how the findings from the social and language processing studies will follow each of these separate parts of the review, and each summary will attempt to evaluate how the eye movement patterns has advanced understanding of cognitive processing in ASD. Specifically, we will evaluate whether there are any consistent patterns of eye movements, that reveal subtle processing differences across the language and social processing domains that could account for the well documented characteristics of ASD in everyday communication.

## 2. Eye-Movement Studies and Language Processing in Autism

Language development and processing has been widely reported to be different for autistic individuals, relative to typically developing (TD) individuals. For example, autistic children may have a delayed onset of language production in comparison to TD peers and may demonstrate differences in pragmatic and higher-level language processing throughout adulthood [6,7]. Such differences have clear potential to contribute to social and communicative difficulties that are characteristic of ASD. Below, we summarise the eye tracking research that has examined the cognitive underpinnings of language processing differences in ASD, and in this section the summaries are presented according to the different paradigms adopted for language investigations in this field. 

## 3. Referential Word Learning Paradigms 

Individuals on the autism spectrum are often delayed in the development of oral language and until recently, this was considered a key component of autistic disorder [3]. Research has suggested that atypical social attention and pragmatic understanding in ASD may influence language acquisition. For example, the importance of interlocutor engagement and joint attention, to support the mapping of novel words to unfamiliar objects has been well documented [8,9]. Several researchers have used eye tracking to examine exactly how autistic children allocate attention during language learning contexts. These experiments typically involve a *learning phase* whereby children with ASD are exposed to novel (and familiar) words. During this phase, eye movements are monitored to compare how children with and without ASD attend to the available information. A *test phase* follows the learning phase to examine word learning which is assessed in different ways in different studies e.g., pointing or naming tasks. Word acquisition rates appear to be similar between ASD and TD children in referential learning contexts, but eye movement data has revealed subtle differences in the routes through which individuals with ASD attain this information.

**Figure 1:**
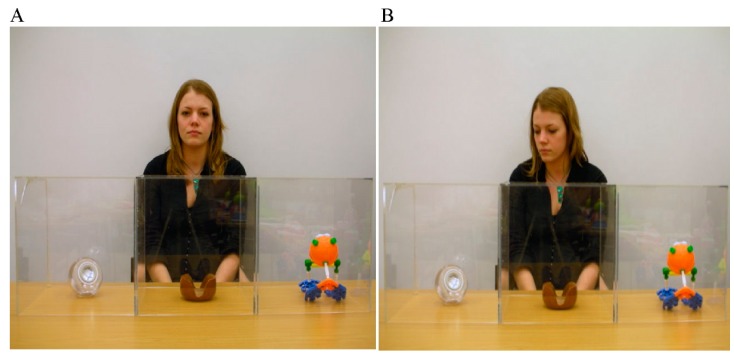
Example of the word learning paradigm used by Norbury et al [10]. Reproduced with permission from Elsevier. Image (**A**) is an example of the gaze neutral condition and image (**B**) is an example of the gaze bias condition.

Most predominantly, research has focused upon examining how gaze cues support word learning in young autistic children. Norbury et al. [10] asked children (aged 6–7 years) to view dynamic scenes of an interlocutor and three objects whilst concurrently hearing novel (and familiar) words. Interlocutor gaze was either biased (directed towards the referred object) or neutral (directed towards the camera). An example of the experimental procedure can be seen in Figure 1. Participants were tasked with clicking on the object that matched the spoken form. 

Both TD and ASD participants used gaze direction to accurately identify target objects. This was demonstrated by faster and more accurate responses when gaze was biased, in comparison to when gaze was neutral. In addition, the interlocutor’s face initially captured the attention of both ASD and TD children. However, eye movements revealed qualitative differences in the use and understanding of gaze direction. TD children fixated the face more than ASD children in the biased condition and TD children made more gaze-object contingent looks (fixations upon the target immediately following a fixation upon the interlocutor’s face) in the gaze-biased condition, in comparison to the neutral condition. For ASD children, gaze-object contingent looks were not modulated by gaze condition. Furthermore, autistic children demonstrated higher initial recall of phonological information, but reduced recall of semantic features, relative to TD participants. Over time these recall rates remained relatively stable for ASD participants, but TD participants’ performance increased for both phonological and semantic recall. The authors suggest that the autistic children’s understanding of the social intention of gaze direction may be less well developed than TD peers, and that gaze may instead be used as an associative learning cue. This difference in the use of eye gaze as a social reference cue to support word learning has the potential to interfere with language learning and development. Although initial word learning rates may be similar in children with ASD, the routes to attaining this information may be qualitatively different, with sound potentially being prioritised over semantic information and social cues in ASD. 

Since Norbury et al. [10] which was the first study to examine referential word learning using eye tracking, several studies have extended these findings using similar paradigms. Akechi et al. [11] used a word learning paradigm to examine attention during referential word learning in Japanese speaking children with ASD (aged 6–11 years) and Tenenbaum et al. [12] examined word learning in young children who were just starting to produce language (aged 2–5 years). In contrast to Norbury et al. [10], neither Akechi et al. [11] nor Tenenbaum et al. [12] reported group differences in fixations to the interlocutor’s face or in saccades made from the face towards the target. Akechi et al. [11] used a schematic image of a face, which may have influenced the children’s willingness to direct their attention to this stimulus, and Tenenbaum et al. [12] reported that attention allocation towards the eye and mouth region, whilst the interlocutor verbally labelled objects, was predictive of faster recognition during the test phase for autistic, but not TD participants (when adjusting for age). Akechi et al. [11] reported that both TD and ASD children fixated the target more than the distractor in a ‘follow in’ condition (when the schematic face followed the child’s gaze to fixate the same object), but that only TD children fixated the target more than a distractor in a discrepant condition (when the schematic face was fixating a different object to the child). Fixations upon both the target and distractor were approximately equal for ASD participants in the discrepant condition. This suggested the autistic children may not have identified that the object the face was directing its gaze towards was ‘special’. 

Both Akechi et al. [11] and Tenenbaum et al. [13] extended these findings to examine whether word learning in ASD could be improved if alternative methods were used to direct attention towards a target object. Akechi et al. [11] made target items more salient via movement and this increased word learning for both ASD and TD groups, with differences in attention allocation between groups for the discrepant conditions disappearing. Tenenbaum et al. [13] demonstrated that word learning can be improved in both TD and ASD groups by explicitly increasing the likelihood of attention allocation to both the interlocutor’s mouth and to the target object, by asking the interlocutor to hold the target item near to their face. These results indicate that alternative non-gaze methods to increase target saliency have the potential to support and improve referential word learning, for both autistic and TD children. 

In contrast to the studies reported above, Lucas and Norbury [14] did not examine the use of gaze direction, but instead, investigated whether orthographic information supports word learning in children with ASD (aged 7–12 years). Participants heard words and concurrently viewed pictures of these words that either included or excluded the orthographic form (if excluded, the ‘word’ area of the screen remained blank). During the learning phase, gaze behaviour was very similar between TD and ASD groups with only subtle differences being detected. These subtle differences included increased duration (but not proportion) of fixations upon the target image for TD participants in the orthography present condition, in comparison to ASD participants. In addition, in the orthography absent condition ASD participants made a higher proportion of fixations upon the blank region where a word could be presented, even though there was no word present. This finding was interpreted to suggest that autistic children may have relied more heavily on orthographic form during word learning, and support for this reliance effect was mirrored in the off-line learning data which demonstrated that autistic participants had higher levels of facilitation, than TD participants, for orthography present conditions. In addition, superior phonological coding was found for children with ASD relative to TD children in the initial test phase, supporting Norbury et al.’s. [10] work. However, in contrast to Norbury et al.’s [10] work, no differences in semantic learning or consolidation were detected. One factor that could explain the reason for the inconsistencies between these two studies is that, in the Lucas and Norbury [14] study learning was examined following a 24-h break, and this length of time is considerably shorter than the four week break used by Norbury et al. [10]. It is possible that either orthography facilitates consolidation for participants with ASD, or that 24 h is not sufficient to detect group differences in longer-term consolidation. 

### Key Findings

- Overall, referential word learning rates in children with ASD without additional language impairment appear to be comparable to TD controls;
- Children with ASD may rely more heavily on phonology and orthographic form during word-learning contexts;
- Eye tracking has revealed mixed findings in relation to the use of gaze information to support word learning in ASD. Some studies report subtle differences in the use of gaze cues, whereas others do not;
- Referential word learning may be improved for ASD and TD children if orthography is present and if attention is directed to target objects using extra cues in addition to gaze direction e.g., movement.

## 4. Intermodal Preferential Looking Paradigm

The intermodal preferential looking paradigm (IPL) has also been used widely to examine language processing in young children. Typically, in this paradigm children freely view two videos as they concurrently hear spoken language [15]. Children that comprehend the spoken language accurately, typically direct their gaze towards the video that reflects the meaning of the concurrently spoken language. The advantage of IPL is that the paradigm provides information as to the incremental processing of spoken language for very young children, without the requirement of additional task demands. However, since gaze direction and latencies are typically coded offline, this paradigm does not provide the level of temporal and spatial precision that can be gained from video-based eye tracking systems. Such precision might be important if there are subtle group processing differences that could account for the failure to develop certain language skills that could foster language communication abilities in ASD.

The IPL paradigm has demonstrated that the time-course of familiar word identification and the noun learning bias are intact in young children with ASD [16,17]. This indicates that early lexical acquisition and processing occur similarly in ASD and TD children. In contrast, Tek et al. [17] found no evidence of the shape learning bias for children with ASD (aged 2–3 years) across four time points over a year, relative to TD children. This bias refers to children’s mapping of a novel word to an object’s shape, as opposed to other features (e.g., colour) and provides insight into the development of semantic categorisation. This effect was reported to be related to the severity of ASD symptoms which suggests that the categorisation mechanisms adopted to learn new words may be different in autistic relative to TD peers, or that the onset with which autistic children adopt this mechanism is delayed. Note however that no difference in vocabulary size was detected between TD and ASD participants at any time point, indicating that an omission to use a shape bias does not appear to have influenced the speed of language acquisition in ASD. In addition, this finding should be interpreted with caution given that oral language skill was not measured and may have confounded these effects. 

Beyond lexical processing, IPL has demonstrated evidence of intact grammatical processing for young children with ASD in the form of comparable subject-verb-object (SVO) structure comprehension [16,18], aspect morphology/tense processing [19], and syntactic bootstrapping [20] *Wh*-questions (e.g., where, who, when, what) are minimally used by children with ASD and provide grammatical and pragmatic challenges. These questions often deviate from SVO structure, involve an understanding that the *wh* word represents information absent from the sentence, and involve the speaker assuming the knowledge of another prior to producing a *wh*-question. IPL has been used to demonstrate that the onset of *wh*-question comprehension precedes production for both TD and ASD children, but that the onset of this stable comprehension is chronologically delayed for autistic children, in comparison to TD counterparts, at approximately 54 months compared to 28 months [21]. Note though that this effect occurs when ASD and TD children have similar levels of language, and, onset of stable comprehension at the individual level appears to be related to early linguistic and pragmatic competence [18]. Therefore, it would seem that the developmental delay in language production, coupled with pragmatic challenges, contribute to differences in language use which has the potential to feed forward and impact upon everyday communication difficulties in ASD. 

### Key Findings

- Lexical acquisition and processing appear to be similar between TD and ASD participants;
- Autistic children may not adopt a shape learning bias, or they may be delayed in the development of this bias, but this does not appear to impede language acquisition;
- Comprehension of basic syntactic form develops similarly in young children with and without ASD;
- Comprehension and production of *wh*-questions is chronologically delayed for autistic children relative to TD children and is likely a result of delayed language development and pragmatic challenges.

## 5. Visual World Paradigm

The visual world paradigm [22] requires participants to listen to spoken sentences (e.g., *The boy will eat the cake*) whilst concurrently viewing a visual scene that typically includes four objects (e.g., cake, boy, ball, bike). One of these objects will always match a word contained within the sentence, and that object can be predicted by prior linguistic content, such that participants make anticipatory eye movements towards this object before it has been spoken. Given the established relationship between eye movements and on-going linguistic interpretation [2], the speed with which sentences are processed can be readily observed. Studies employing this paradigm have demonstrated that individuals with ASD incrementally process verb information and make on-line predictions about the constraints of upcoming linguistic input. For example, similar proportions of anticipatory eye movements made towards a target item (e.g., hamster) upon hearing a biased verb (e.g., stroked) in comparison to a neutral verb (e.g., moved) have been reported for English speaking adolescents [23], young children [24], and Mandarin speaking children with ASD [25] in comparison to TD samples. 

Hahn, Snedeker, and Rabagliati [26] used the visual world paradigm to examine the on-line processing of ambiguous words in ASD (e.g., star) when embedded in contexts that suggested that either the dominant (star in the sky) or the subordinate (movie star) meaning should be accessed. Participants with and without ASD both demonstrated evidence of using early sentence context to inhibit inappropriate ambiguous word meanings. Specifically, both groups showed evidence of reduced anticipatory eye movements towards an object that reflected the dominant word meaning within the first 500 ms of hearing a word, when the context was biased towards the subordinate meaning. This paradigm has also demonstrated that children with ASD use prosody to disambiguate syntactic ambiguities as efficiently as TD peers [27]. Moreover, Bavin et al. [24] found that autistic children were as effective as TD children in the use of context to detect and override an initial implausible sentence interpretation for sentences that contained ambiguous preposition phrases (e.g., The girl cut the cake with the candle). What should be evident from these studies is that when eye tracking tasks are used to examine incremental processing of language as speech unfolds, autistic children and adolescents do not appear to differ to TD comparison groups in the processing of context to predict, disambiguate, or update interpretations of incoming auditory information. This finding contradicts cognitive theories of ASD that suggest that global contextual processing may be atypical in ASD [28] and indicates that communication difficulties in ASD are unlikely to be related to autistic individuals failing to process linguistic context or compute on-line predictions about up-coming input. Note that these null group effects are reported when there is no requirement for a social response. Social demands may interfere with the efficiency of such processing in everyday communication. 

### Key Findings

- Visual world experiments demonstrate that children and adolescents with ASD use context and verb information to incrementally predict upcoming linguistic information;
- Visual world experiments demonstrate that children and adolescents with ASD disambiguate lexical and syntactic information at a similar time-course to TD comparison groups.

## 6. Listening Whilst Looking Paradigm

This paradigm is similar to the visual world paradigm in that it requires participants to look at images whilst hearing spoken language; however, in the listening whilst looking paradigm explicit auditory instructions are given to fixate one of the images. Bavin et al. [29] used this paradigm to examine how ASD symptoms severity influences the efficiency of lexical access. Children (aged 5–7 years) viewed four images including a target (e.g., boy), a phonological competitor (e.g., a box), and two unrelated distractor objects whilst concurrently hearing ‘where is the *boy*?’ Children with more prominent ASD symptom presentation were less likely and slower to fixate the target, compared to TD children and to ASD children with less prominent symptom presentation. Moreover, when autistic children did fixate the target, they shifted attention away from the target more quickly than TD children. These effects were not modulated by IQ and nor by oral language skill, and the authors suggest that this may indicate that the efficiency of lexical access is influenced by ASD symptom severity. In contrast, Venker, Eernisse, Saffran, and Weismer [30] who also adopted the listening-whilst-looking paradigm, found on-line accuracy and eye movement latencies for familiar words to be highly variable in children with ASD (aged 3–5 years old), following hearing questions such as “*Where’s the __? Do you see it*?” but the findings were not associated with autistic symptoms. Instead, on-line accuracy was primarily associated with language competence. Note that the nature of this task involves participants comprehending and responding to *wh*-questions and, as described earlier, these types of questions (with high pragmatic and grammatical demands) are known to be particularly challenging for children with ASD [18,21]. The differences in task demands may explain why autistic symptomology was found to be predictive in Bavin et al. [29], but not in the Venker et al. [30] study, where prompts were not exclusively *wh*-questions. 

This paradigm has also been extended, beyond investigating aspects of lexical processing, to examine incremental semantic and syntactic processing when listening to sentences that contain a noun modification (e.g., “*Look at the blue square with the dots*”) whilst simultaneously viewing four items on a screen, including the target object (e.g., a blue square with dots), a competitor object (e.g., a blue square) and two distractor objects [31]. The findings from that study show that both ASD and TD children fixated target and competitor objects more than distractor objects, upon hearing the noun phrase, and both groups fixated the target more than competitor when hearing the modifying information (e.g., with the dots). However, group differences revealed that the ASD group were slower to fixate the target, and that they had a lower proportion of looking time to the target overall, in comparison to TD participants. What the listening whilst looking experiments demonstrate is that whilst individuals with ASD can correctly comprehend auditory information and match this to the visual display, there are subtle differences in the speed of this processing in children with ASD, and, depending upon the pragmatic demands of a task, this efficiency may be related to symptom severity. 

### Key Findings

- The listening whilst looking paradigm demonstrates that the efficiency of lexical access may be mediated by symptom severity in ASD children;
- ASD children accurately process and comprehend noun modifiers; however, the time-course of this processing appears to be less efficient;
- The pragmatic demands of the listening whilst looking paradigm should be considered when interpreting findings.

## 7. Reading Paradigms

Reading skill is highly variable in ASD [32] and is determined by normative factors associated with reading (e.g., oral language, word decoding), in addition to ASD-specific higher-level language processing differences [33]. In general, research reports performance outcomes for reading comprehension tasks to be reduced in ASD in comparison to what would be predicted by age, IQ, or decoding skill [34,35]. However, the underpinning processing differences that contribute to comprehension differences in ASD remain unclear. In recent years, there has been an increase in the use of eye-tracking experiments designed to address this question. Typically, eye tracking tasks that examine reading, involve participants silently reading text on a monitor at their natural rate (typically one sentence or one small passage at a time). To detect word-level gaze behavior, reading paradigms are completed in very controlled environments, using head-stabilized tracking systems with high spatial and temporal accuracy, and involve attaining very precise calibrations typically within 0.25–0.50° accuracy. Note that the samples recruited in the studies reported below are predominantly adults that have received diagnoses of Asperger syndrome and, therefore, did not present with early language delay as children. As such the samples reported below may have fewer challenges associated with language than those diagnosed with autistic disorder (note that Asperger syndrome, autistic disorder, and pervasive developmental disorder were replaced in the most recent version of the DSM with a single diagnosis of ASD). 

Studies that have examined the time course of low-level linguistic processing during reading have demonstrated comparable text processing between TD and ASD readers. For example, Howard, Liversedge, and Benson [36] demonstrated typical frequency effects (low-frequency words are fixated for longer than high-frequency words) for readers with ASD; Caruana and Brock [37] demonstrated typical predictability effects (fixations upon words that are predictable based upon previous sentence context are fixated for less time that unpredictable words) for TD individuals with high autistic traits; and Davidson and Weismer [38] demonstrated expected subordinate bias effects when processing ambiguous words for ASD participants (which were concurrently presented with auditory stimuli). In addition, Howard et al. [36] demonstrated that syntactic parsing preferences and the speed of recovery from syntactic misanalysis when reading sentences that contained ambiguous prepositional phrases was also comparable between TD and ASD adult readers. Thus, the time course of on-line lexical and syntactic processing during sentence reading appears to be comparable between TD and ASD adult readers. 

There is also a body of work that has focused upon using eye-tracking methodology to examine higher-level aspects of reading. Given that these higher-level linguistic processing tasks are where differences in performance outcomes are predominantly reported [33], one would expect there to be differences in the eye movement measures between ASD and TD readers. However, many similarities between ASD and TD readers for these higher-level reading processes have been found when adopting this paradigm. For example, readers with and without ASD have been reported to demonstrate comparable irony processing [39], comparable counterfactual processing [40], comparable and even superior counterfactual emotion processing [41], comparable anomaly detection in real and fantasy worlds [42,43], and comparable co-referential processing [44]. 

Where differences are reported, these are subtle, and are almost exclusively reported for the reading of texts that require inferential processing. For example, Sansosti, Was, Rawson, and Remaklus [45] asked adolescents with and without ASD to read vignettes that evoked a causal inference. They reported more fixations, longer fixation durations, and more regressions back through the texts for the individuals with ASD in comparison to a TD comparison group. What these differences seem to indicate is that the processing of such text required more effort for ASD readers. However, Sansosti et al.’s [45] analyses were restricted to global eye movement measures which were not target related, but rather, the measures were calculated across the entire vignettes. This makes it more difficult to identify the source of such reading disruption, since we do not know when or where this occurred. However, it would seem likely that the disruption observed in the ASD group could be related to the inferential processing that the texts required. 

**Figure 2:**
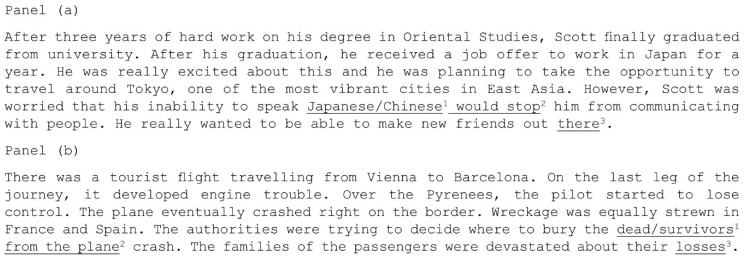
Example of the passage level (**a**) and sentence level (**b**) anomaly stimuli used within Au-Yeung et al.’s [47] experiment. Reproduced with permission from SAGE.

In a later study, Micai, Joseph, Vulchanova, and Saldaña [46] examined inferential processing more directly by asking participants to read texts that required an inference to be formed, and then analysing eye movements for localised areas of these texts, areas that should have evoked the inference processing. Critically, TD readers demonstrated longer processing times upon portions of text where inferences were formed. The ASD participants in Micai et al.’s [46] experiment had longer gaze durations, in comparison to TD readers upon the critical words that informed the inference. In addition, ASD readers regressed back through the texts to words that supported and further informed this inference on a higher proportion of trials, in comparison to TD readers. Importantly, no differences in comprehension outcomes were detected. This suggests that ASD readers form inferences on-line during reading, but that inferential work specifically may require more effort for ASD readers relative to TD controls even when IQ and language skills are closely matched. Although the mechanistic explanation as to why inferential processing of this kind may be atypical in ASD remains unclear, two studies have provided some insight into why this atypicality may exist. Firstly, in a study where detection of implausibilities could only be successful if these were evaluated against situational world knowledge, it was reported that such detection is delayed in ASD relative to TD readers [43]. The implication from this finding suggests that the processing of world knowledge during reading, which is often necessary for inferences to be formed, may be less immediate in ASD. It is important to note, however, that more recent experiments that use tasks and stimuli which require world knowledge about fictional characters to be inferred, report an absence of group differences in this area [42]. Therefore, the suggestion that inferential processing differences are related to situational world knowledge processing, and are inherent in ASD, requires further investigation. The second study [47] reported that individuals with ASD were quicker to detect sentence-level anomalies (where the anomaly could be detected by reading the sentence in isolation) in comparison to paragraph level anomalies (where the anomaly could only be detected if the global context of the paragraph had been formed), compared to TD readers (see Figure 2 for an example of these stimuli).

**Figure 3:**
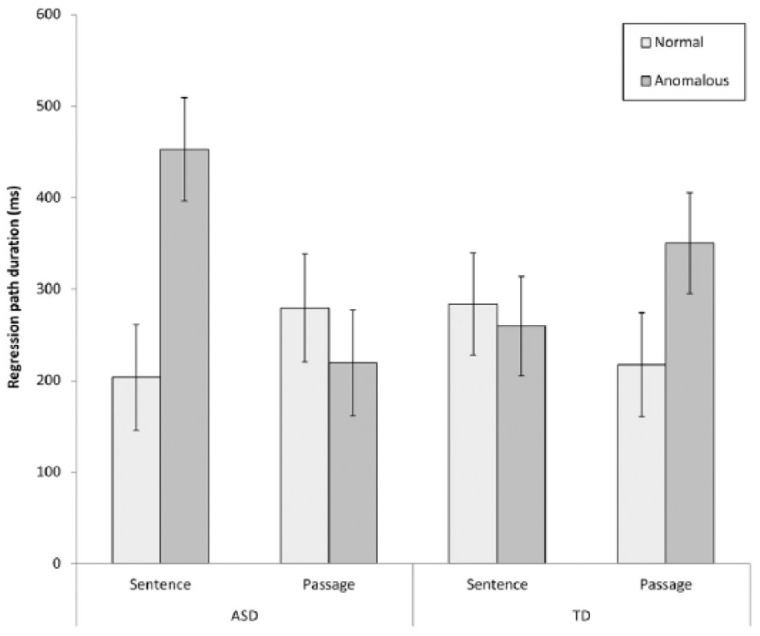
Group and anomaly type interaction for regression path durations reported by Au-Yeung et al. (2017) [47]. Reproduced with permission from SAGE.

ASD readers were slower to begin to resolve passage-level anomalies, relative to sentence level anomalies, and relative to TD readers, as evidenced by regression path times (time from when a region is first fixated until a reader progresses to fixate information to the right of this region). This interaction is displayed in Figure 3 below and suggests that there may be broader differences in the time-course with which ASD readers integrate sentence meaning within discourse representations. Given that the integration of global information into the discourse model is often necessary for inferential processing, time course differences could potentially contribute to inferential and comprehension difficulties previously reported for autistic readers. 

An unexpected yet consistent finding in studies that have used eye tracking to examine reading in adults with ASD is that autistic readers engage in a higher proportion of re-reading, in comparison to TD readers, e.g., [39,43,44,47]. It has been suggested that the re-reading in ASD could reflect a ‘cautious’ reading style [36]. A lack of any modulation of re-reading by text type (e.g., individual experiment conditions) and the finding that re-reading appears to be present even when ASD readers demonstrate expected first-pass reading effects in the eye movement record, suggests that the re-reading in ASD is unlikely to be a result of linguistic processing differences. However, the exact cause of this behaviour remains to be investigated. Since re-reading occurs for single sentences, short (three line) paragraphs and for longer texts, and since it reflects a propensity to ‘go back’ and re-read after having read through the text once in entirety, it is not yet clear whether this behaviour is necessary for full comprehension of what has been read, whether it reflects one arm of the diagnostic criteria for ASD (repetitive behaviours), or whether it reflects a strategy for coping with comprehension questions.

### Key Findings

- On-line lexical and syntactic processing is comparable during reading in ASD and TD adults;
- When adopting reading paradigms, TD and autistic adults do not differ in a range of higher-level language processes;
- The time-course of on-line inferential processing may require more effort for autistic adults;
- ASD readers tend to re-read texts more than TD readers.

## 8. Language-Processing Summary

Eye-tracking paradigms provide a valid way to measure incremental language comprehension in ASD with minimal task or response demands. Throughout all paradigms any apparent processing differences *predominantly* reflect quantitative delays, as opposed to qualitative deviances. Temporal processing differences, as revealed by eye movement measures, appear to be present when pragmatic and higher-level linguistic demands increase. Time-course differences in language comprehension at any level have clear potential to impede the fluidity and reciprocal nature of everyday communication, and such differences may be exacerbated or onset by increased pragmatic demands associated with conversational exchanges. Any such delays, even if very subtle, may result in autistic individuals comprehending language input later than TD comparison groups, particularly when rate of delivery cannot be controlled. The repeated reading in ASD may be needed for full text representation or comprehension, but in everyday communication there is no opportunity to ‘go back’ and resample what has been said. Thus, the eye movement studies investigating language processing over the past 10 years have provided insight into how subtle (temporal) processing differences might impact in everyday communication difficulties in ASD.

## 9. Eye-Movement Studies and Social Processing in Autism

Difficulties in social interaction and communication are a key characteristic of ASD and atypical social processing has been consistently reported in numerous studies [3,48,49]. Using eye-tracking technology, researchers have monitored and analysed the eye movements of infants, toddlers, children, adolescents and adults with autism, for a diverse range of social stimuli or social contexts, to examine the nature and the time course of any on-line processing differences between TD and ASD individuals. Using a variety of paradigms, numerous eye movement studies have provided ample evidence of how individuals with autism detect, attend to and show understanding of social information, and implicit social cues. The findings from these studies have revealed to some extent the relationship between visual attention to social information and ASD specific behaviour in everyday communication.

## 10. Joint Attention Paradigms

Joint attention is an important aspect of early social development and eye gaze can be used as a salient cue to help people understand the social world and to predict the actions of others. Several studies have investigated whether autistic individuals can engage in joint attention by monitoring eye movements, and results have shown that there are differences in joint attention and gaze-following behaviour in children on the autism spectrum. Gaze-following is a precursor to joint attention and has been found to be atypical in young children with ASD. As well as investigating eye gaze, some studies have also examined head following in ASD. For example, Vivanti, Trembath, and Dissanayake [50] investigated visual attention responses to head turns, and the findings showed that young children with autism (aged 46 months on average) responded less to turning heads compared to a TD group, with no significant increase in attention to the face and the target in head turning conditions relative to neutral conditions. Thorup et al. [51] showed that in a real interaction, compared to low-risk infants, infants (aged 10 months) who were at risk for autism tended to rely more on head turns, than on isolated eye gaze shifts, to follow another’s gaze direction. One potential reason for this result may be that the cueing effect of eye gaze is less salient than a head-turning cue, such that autistic children may find it difficult to follow eye gaze in the presence of a turning head, or, that the development of gaze following is delayed in ASD children relative to TD children. There is also some evidence to suggest that engaging in joint attention can be explicitly improved in children on the autism spectrum. For example, Krstovska-Guerrero and Jones [52] reported that, following a 3–9 week training intervention in eye gaze behaviours, all toddlers with ASD mastered eye gaze following. Moreover, Navab, Gillespie-Lynch, Johnson, Sigman and Hutman [53] found correlations between responsive joint attention (RJA) to eye gaze as measured by eye-tracking and scores on the Early Communication Scales in infants, which provides evidence for the validity of eye tracking to assesses RJA, and also shows that joint attention is related to communication abilities in ASD.

When examined in older children, joint attention behaviours appear to have developed in ASD, and any group differences are more subtle than would be expected based upon the stark differences in early head and gaze following reported above. Swanson and Siller [54] used an adapted attentional cueing paradigm, whereby faces were presented at the central point of the screen with an object presented in peripheral vision. The eye gaze of the central face was shifted either to the direction of the object (congruent) or to the direction opposite from the object (incongruent). Participants without autism looked longer towards the object in the congruent condition, relative to the incongruent condition, as evidenced by increased early (first fixation duration, FFD) and later (total fixation time, TT) stages of processing. The ASD participants showed similar effects for TT, but not FFD. Similarly, Falck-Ytte, Thorup and Bölte [55] revealed a weaker initial processing bias for attended objects in young children with ASD compared to TD children and children with developmental delays. Using more naturalistic social stimuli, it has also been demonstrated that adolescents with ASD show intact global processing of eye gaze, but that the time-course with which gaze was followed is less immediate for adolescents with autism, relative to TD adolescents [56]. Furthermore, Riby et al. [57] found that, when given an explicit instruction, participants with autism showed no tendency to follow the cue and to increase fixations on the location of a gazed-at target. Together, these studies indicate a subtle difference in the initiation of gaze following behaviour in ASD.

The above studies examined the response to joint attention (RJA) cues in children with ASD. However, a recent study also examined the spontaneous initiation of joint attention (IJA) [58]. When toddlers were watching a person shift their gaze to one target, no group differences were found in response to the attentional allocation towards faces and objects (target and non-target), and to the transitions between the face and target. However, in IJA conditions, participants with ASD showed atypical fixation patterns. Toddlers with ASD looked longer to the face and made more transitions from the target/related object to the face but less transitions between the non-target/unrelated object and the face, compared to the TD group. Moreover, higher levels of atypical eye movement transitions in IJA conditions in autism were associated with more severe ASD symptoms. Based on the atypical viewing patterns in the IJA condition exclusively, it seems that JA differences in ASD may be related more predominantly to differences in initiation of JA, as opposed to the understanding of JA initiated by others.

A further early developing social behaviour related to JA is imitation, and, studies have revealed atypical imitation in ASD [59,60]. For example, Vivanti et al. [59] asked participants with autism to view short videos showing a goal-directed action being performed by an actor whose gaze was either directed towards the viewer or averted from the viewer. Results showed that for TD children, direct eye gaze from the person in the video increased participant fixation time to the person, and enhanced the performance of spontaneous social imitation. In contrast, for children with autism, there was no difference in social attention and social imitation accuracy between the direct and averted eye-gaze condition. This finding indicates that reduced imitation in ASD may be associated with reduced attention towards a person’s communicative signals. Since imitation has been reported to be reduced in autism for direct eye gaze conditions, but not for averted eye gaze conditions [60], it could be inferred that the use of direct gaze may hinder imitation in ASD. The relationship of atypical visual processing for salient social cues (direct eye gaze or head turning) and poorer imitation performance in autism, as revealed in these two studies [59,60], support the hypothesis that subtle differences in social processing have the potential to impact in social interaction behaviours in everyday communication in autism, and that hypothesis is also supported by many other studies [61,62,63].

### Key Findings

- Infants and young children with ASD, or at risk of ASD may rely more heavily upon head turns, as opposed to eye-gaze shifts as social cues;
- Children and adolescents with ASD engage in joint attention behaviours, but the initial onset of gaze following is delayed, relative to that observed for TD individuals;
- Similar to what is found for TD children, engaging in joint attention behaviours supports social development in ASD;
- Children with ASD show a different pattern of attention when initiating joint attention, in comparison to TD children;
- Direct gaze may not facilitate imitation in ASD and may even reduce spontaneous social imitation behaviour.

## 11. Free Viewing Paradigms for Faces and Social Scenes

Attentional biases to social information play a fundamental role in shaping typical development of social cognition and behaviour in humans. However, a number of studies which have utilized the free viewing paradigm report both typical and atypical social attention in ASD, relative to TD individuals. In a free-viewing paradigm, participants are presented with a visual stimulus which they look at without instruction, as their eye movements are monitored. Findings from this paradigm have reported that individuals with autism show decreased spontaneous attention to faces, and to people [55,57,62,64,65,66,67,68,69,70,71,72,73,74], and there is a reduced preference to look at the eyes or mouth regions when attending to faces [57,74,75,76,77,78,79,80,81], regardless of age of autistic individuals. Several studies have also revealed that autistic individuals show an attentional preference for less-salient social elements (e.g., bodies) and objects (e.g., backgrounds) in scenes [75,82]. This attenuated social attention in autism is mainly evidenced by two categories of eye movement measures, which are shorter viewing time (or the percentage of viewing time) and fewer fixations on social items. These two eye-movement measures are thought to reflect general processing of the social world, and the findings from the above studies seem to suggest that there is a reduction in general attention towards socially relevant information in ASD. However, and in contrast to those findings, other studies have reported evidence for preserved social orienting in autism. For example, in some studies individuals with autism have been reported to show the same probability as TD controls to execute their first saccade (rapid eye movement from one location to another) to social stimuli [83], and they have been reported in other studies to take the same time as TD viewers to initially direct and move their eyes to the social stimuli [84,85]. Furthermore, once the social stimulus has been fixated, the ASD group then shows intact social engagement (fixation duration), which is equivalent to counterpart TD controls [86,87,88,89,90,91,92]. Dicriscio et al., [93] adopted the anti-saccade paradigm [94], where the task is to look to the opposite direction of a peripherally presented target, to examine attentional control in autism for social (happy faces) and non-social stimuli (e.g., cars, shoes). This study found no differences in saccadic inhibition for social stimuli, as indicated by similar error rates (eye movements directed towards a social stimulus instead of away from it) in both groups, providing further evidence that social information can capture initial attention in ASD.

Several other studies report evidence for subtle differences in spontaneous social orientation in autism, with no atypicality reported for overall attentional processing of social cues [95,96]. For example, Freeth, Ropar, Mitchell, Chapman and Loher [95] found that although adolescents with autism took longer to first fixate on the person presented in social scenes, compared to age and IQ (full IQ and verbal IQ)-matched TD adolescents, the total fixation time measure indicated that both groups attended to social information similarly overall. Using the preferential looking paradigm, Guillon et al. [96] examined attentional biases to face-like objects paired with inverted face-like objects, which are usually not perceived as face-like. The results showed that young children with autism were less likely to direct their eyes initially to the upright face-like objects relative to an age matched TD group, but overall they spent a similar amount of time fixating on the upright face-like objects as the TD group. This result is consistent with the finding of Freeth et al. [95] for social scenes stimuli and does not suggest there is any overall general social viewing deficit in individuals with autism. The one subtle group difference that appears to exist from these studies is a reduced social prioritization in ASD participants, in relation to where their first saccade is directed.

Several studies have tried to provide detailed analyses of eye movement patterns for dynamic social information, by adopting analyses approaches which are different from the classic region of interest (ROI) analysis method (as reported for the above studies). These alternative analyses examine both the temporal and the spatial eye movement patterns simultaneously, across the whole duration and display of dynamic social interaction videos in ASD [97,98,99]. These studies consistently report that people with autism do not follow social events in dynamic interactions as efficiently as their TD peers. When attending to video clips showing several people conversing with each other, or when people in video clips speak to the audience in turn, participants within the TD group showed similar temporal–spatial gaze patterns, indicating that the TD participants tend to look at the same place in a specific moment in time. However, ASD participants did not show this viewing pattern. Further frame-to-frame analyses examined the potential causes of this atypicality and found that individuals with autism fixated less on people, accompanied by increased attention to non-social stimuli in the videos. Critically, participants with ASD tended to shift their attention from the speaking person ahead of the TD group [97,98]. This viewing pattern is observed in both ASD children and in ASD adults and demonstrates that ASD viewers are not processing the information in the same way as their counterpart TD peers. It is not known from these studies whether the ASD participants are similar to each other in the way they allocate attention during these tasks, but this would be worthy of investigation. Another finding has shown differences in how TD participants sample the information in these tasks. It appears that young TD children prefer to monitor the mouth rather than the eyes, while the TD adults showed a reversed pattern. This finding might potentially help to explain the lack of difference in fixation on eyes or mouth regions between both the TD and ASD participants. In Falck-Ytter et al.’s study [99], participants were presented with videos of semi-naturalistic social interactions between two young children. The results showed that, compared to the TD group, children with autism had decreased preference to look toward to the person who was likely to guide next interaction.

These findings indicate that children with ASD may be inattentive to social content when attending to dynamic social interactions. This result is consistent with the findings from studies showing inadequate attention to salient social stimuli in complex social scenes or dynamic videos [57,73]. The studies employing dynamic stimuli illustrate how eye movements are able to reveal subtle but potentially significant foundations of social-processing differences in autism, and, failures to follow or act upon available social cues in dynamic conversation may result in failures in reciprocity in every day communication in ASD.

### Key Findings

- Autistic individuals may have decreased spontaneous attention to social information in free viewing paradigms;
- Autistic individuals may have a reduced preference to process eye and mouth regions of faces, relative to TD individuals;
- Autistic individuals show atypical attention to, and processing of, social information in dynamic interactions, relative to TD individuals.

## 12. Circumscribed Interest and Geometric Pattern Paradigms

An increased interest in circumscribed interests (CI, e.g., trains, electronics) or geometric patterns processing has been offered as an alternative account to illustrate social-processing characteristics in autism [100,101,102,103,104,105,106]. Results from these studies suggest that the presence of CI stimuli attract more attention in autism [100,101], resulting in decreased viewing time on social images [102] compared to when CI stimuli are present. Using paired dynamic social videos and geometric pattern displays, studies [103,104] found that toddlers (14 months) with autism spent significantly less time viewing social images compared to toddlers without autism. Furthermore, more than 69% of total time spent looking at geometric patterns could predict an autism diagnosis with 100% accuracy [103]. Moore et al. [105] investigated this issue with a larger group of toddlers and replicated the results from Pierce et al. [103], and this preference for geometric stimuli continues from infancy into childhood [106], with autistic children making fewer fixations on social interaction videos when these are presented with dynamic geometric stimuli simultaneously. From these studies it appears that the viewing pattern of social stimuli in autism depends on the type of competing non-social stimuli that are also present. The high level of attentional bias to either CI or geometric stimuli may result in reduced social attention in autism, and hence a greater likelihood of relevant social information going undetected.

However, this bias is not equivalent for all individuals with autism. Moore et al. [105] identified a geometric pattern-preference ASD subgroup and a social stimulus-preference ASD subgroup, and showed that, the increased attention to geometric patterns in autism is related with symptom severity, potentially indicating that increased attention to this kind of stimulus can be used as a prognostic tool. This result reveals the important influence of individual differences within the ASD group in relation to social processing. However, it is important to highlight that low-level stimulus features (e.g., spatiotemporal frequency, contrast, and luminance) are often not controlled across stimuli in the experiments reported above, which means that low-level differences in the visual characteristics of stimuli cannot be ruled out as likely contributors to some of the observed attentional differences.

### Key Findings

- Differences observed between TD and ASD children in the visual processing of social information may not reflect atypical social processing per se, but instead increased attention towards alternative stimuli of interest;
- The attentional capture of CI interests and geometric patterns may be associated with ASD symptom severity.

## 13. Stimulus and Task Complexity

A further factor that has been shown to modulate allocation of attention to social information in ASD is the complexity of the social stimuli being viewed [69,76,106,107,108,109]. For example, Hanley et al. [76] found that when faces were presented in isolation, individuals diagnosed with Asperger syndrome spent a similar amount of time fixating faces compared to age and IQ matched TD individuals. However, in social scenes including more than one person, ASD participants showed reduced attention to eyes relative to the TD group. Further evidence comes from Chevallier et al. [69] who found that, compared to an age-matched TD group, ASD children reduced their attention to faces and increased their fixations on objects exclusively for complex dynamic social interaction situations. Using dynamic social stimuli, Chawarska et al. [107] investigated the modulation of social salience on the attentional allocation in toddlers with high risk for autism. The results showed that the high-risk group (who were later diagnosed with autism) attended to social stimuli typically in a goal-directed action condition (an actor was making a sandwich and made no direct eye contact with viewers) and in a moving toys condition (actor directing gaze towards moving mechanical toys) in which the salience of social behaviour was relatively low. In contrast, in conditions involving more salient social cues or interactions, like the dyadic bid (direct interaction from the actor in a video towards the participant) and joint attention conditions (the actor was directing her eye gaze towards the participants and then towards specific objects), ASD toddlers fixated less on the screen, and less on the face and mouth regions of the actor compared to the TD toddlers.

By developing a novel data-driven method of analysing eye movements for dynamic information, Wang et al. [108] investigated the time intervals as to when clinical and non-clinical toddlers looked at the same content in the social video context depicted in the Chawarska et al. [107] study. The results from this analysis showed that in the dyadic bid and in the sandwich-making condition, ASD toddlers had lower converging attention allocation to the same spatial location at specific moments in time, compared to the both a TD and a developmental delay (DD) toddler control group. However, in a moving toys condition, there were no group differences. No difference was found between the DD and TD group in any condition. The finding from this on-line analysis has been interpreted to suggest that ASD toddlers showed atypical gaze patterns in response to social bids. Furthermore, this atypical attention pattern in early life, which seems to have little to do with intelligence, was related to more severe social-affective symptoms, as assessed by the Autism Diagnostic Observation Schedule (ADOS). The eye movement patterns from the dynamic video studies indicate that deficits in social attention from early life might impede the detection of key social information, and thus might adversely impact upon the acquisition of social experience in later development. The effects of different types of dynamic social stimuli [107] coupled with the temporal and spatial analysis of the eye movements during the video presentation [108] have been invaluable in showing that atypical social attention in autism is observed for more complex social situations, and this atypicality in ASD appears to be absent for more simple social situations.

Further support for differences between TD and ASD groups in viewing complex scenes comes from Shi et al. [106] who compared visual preferences for simple and complex dynamic social stimuli in preschool-aged children with and without autism. Each social stimulus was presented with simultaneous presentation of a dynamic geometric pattern. The results suggested that ASD participants viewed social images less than the TD children in the complex social stimulus condition (which included several people playing games), whereas, for the simple social stimulus condition the group difference was absent. Using the same paradigm, Crawford, Moss, Oliver, Elliott, Anderson and McCleery [109] presented participants with social and non-social video stimuli in two conditions (moving towards or moving past the viewer) and found reduced preference to social over non-social videos in ASD only when stimuli were moving towards the viewers.

It is clear from the above studies that stimulus complexity can affect social processing in ASD, but what about task complexity? It is well established that task instructions can affect the allocation of attention to information in scenes [110], and studies that have directly examined the influence of task instructions on social attention in ASD have also found a failure in modulating eye gaze to pre-specified target areas (e.g., eye regions or eye-gazed targets) where they were guided to attend to these under an explicit instruction [57,111]. In a cross- modal study, Grossman, Erin, Teresa and William [112] investigated the modulation of task demand on preferential attention to the auditory-visual (AV) synchronization of speech in children with and without autism. Two videos of a speaker’s mouth were presented concurrently, and these were either synchronized or not synchronized with speech audio. Participants were either asked to view the display freely (implicit task) or they were guided to look at the synchronized speech (explicit task). Grossman et al. [112] found that although both groups increased their fixation duration on synchronized speech in the explicit task condition compared to the implicit task condition, this tendency was reduced in ASD children and this group also looked significantly less to the AV-congruent image, and to the mouth region than their TD peers.

**Figure 4:**
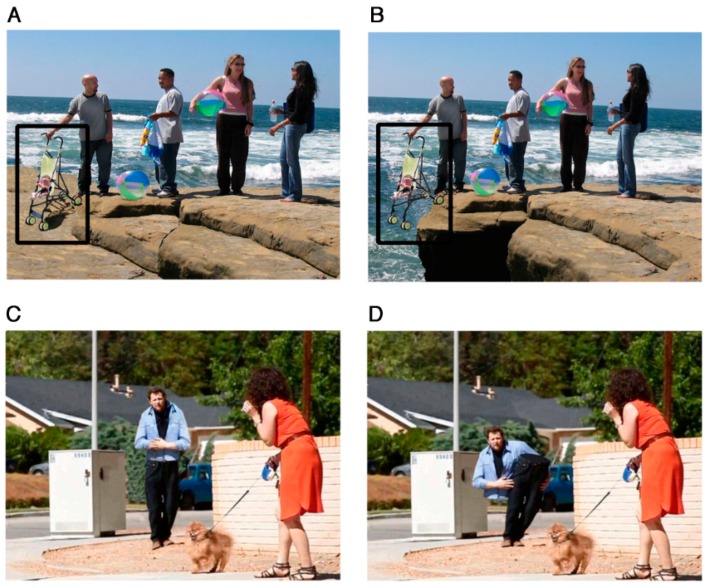
Example of social normal (**A**), social weird (**B**), physical normal (**C**), and physical weird (**D**) stimuli from Benson et al.’s experiment [61]. Reproduced with permission from John Wiley and Sons. The black rectangles represent the target region and were not visible during the experiment.

**Figure 5:**
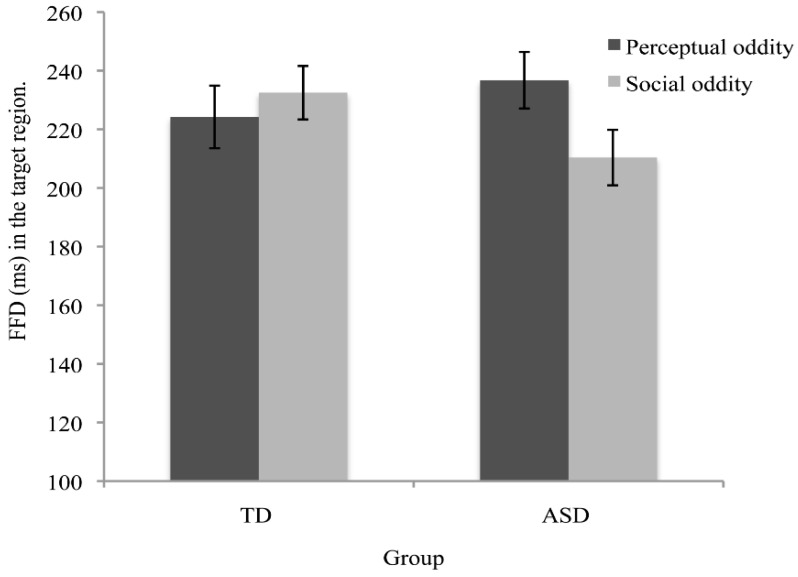
The interaction between oddity type and group for first fixation duration (FFD) detected in Benson et al.’s [61] experiment, indicating a temporal delay in the initial detection of social, but not physical/perceptual, oddities for autistic adults. Reproduced with permission from John Wiley and Sons.

The results from the eye-movement studies reported so far are not entirely consistent and the eye-movement measures reported suggest that there are both similarities and differences in social processing in autism. There are, however, very subtle differences in visual processing of social stimuli in different social contexts [61,113,114]. For example, Benson et al. [61] revealed that, compared to TD adults, when deciding whether a social scene is weird or normal, high-functioning adults with autism fail to recognize the socially weird information during their initial fixation on that information (see Figure 4 and Figure 5). This study showed that the ASD group needed more look backs and longer total fixation time to confirm detection of a socially weird target in the scenes. However, there were no significant differences in detection and processing of a physically weird target event or item, between the ASD and TD groups. This subtle processing difference in the immediacy with which social cues are detected, coupled with the repeated scanning of the target area in autism, may reflect a reduced speed in processing the social contexts depicted in the scenes. The impact of these two consistent findings has relevance for the everyday communication domain in ASD, since any delay in the detection of crucial information would affect the ability to follow what is happening, and in everyday communication, if something is ‘missed’ it is not possible to go back and recheck what has already been sampled.

Limitations in the modulation of attention, according to task demands or stimulus complexity, could be an essential factor in shaping any social-processing differences in ASD, and, could potentially result in inappropriate attentional allocation to salient social cues, such as the eyes, or the eye direction for complex social scenes [72,81,115], which could result in failures to respond effectively or appropriately in everyday communication.

### Key Findings

- The allocation of attention to social information is comparable between TD and ASD participants when social stimuli are relatively simple, for example, include one person;
- The allocation of attention to social information is reduced in autistic children and adults when the complexity of social aspects of the scene increase e.g., include interaction;
- The exact determinants of what make a social scene simple or complex requires further examination and definition;
- In scenes that depict socially odd events autistic individuals fail to immediately detect these on initial fixation.

## 14. Face- and Emotion-Processing Paradigms

Atypical face processing is well documented in numerous studies in autism, and individuals with autism are found to show reduced accuracy in face identification and in emotion recognition [92,116,117,118,119,120]. This appears to be a face specific processing difference, which is absent for object recognition [64,117]. We reported, in earlier sections of this review, the differences in visual attention to faces between autistic and non-autistic individuals. These differences were observed most noticeably for naturalistic or dynamic social contexts. In this section we review the studies that have focused on exploring the visual coding characteristics of faces and face-processing cognition, with an aim to evaluate whether such differences might account for difficulties in social processing in autism.

Snow et al. [117] found that whilst a TD group made more fixations on faces compared to objects during a visual encoding phase, an ASD group did not show this pattern. Subsequently, in a recognition phase, the ASD group performed less accurately for a face-recognition test relative to TD participants. This suggests that reduced fixations to faces in an encoding stage might have influenced performance in the recognition stage for autistic participants. Furthermore, Yi et al. [79,80] have shown atypical face scanning during a memory phase and report that ASD participants had a smaller frequency in scanning different core facial features relative to a TD group. The behavioural data from that study also showed the ASD group to have lower identification accuracy, compared to the TD group. Similarly, Liu, Li and Yi [118] used a variable analysis approach to investigate viewing patterns for faces in autism, and they found less fixation time on the right eye but longer fixation time on the region below the left eye (from the observer’s view) in an ASD group compared to a TD group. It is not clear what this viewing pattern might indicate, and at this stage it is perhaps more important to accept that core facial features are fixated less by autistic participants. This reduced fixation for core facial features also fits with findings of reduced numbers of saccades made between core facial features in ASD, and Ellie, Romina, Jon and Dyer [119] have revealed that performance for face identification is positively associated with the number of saccades made between different facial features. Therefore, the widely found face identification differences in ASD appear to be related to atypical face scanning, and several studies seem to also point to a reduction in fixations on core facial features. Together this pattern of saccades and fixations for autistic individuals may indicate either a tendency to avoid eye gaze, or a reduced propensity to process faces holistically. However, what remains unclear is whether this is a face specific difference, or a consequence of general reduced attention allocation towards social information (such as faces), as reported in earlier sections of this review.

Recently, Evers, Van Belle, Steyaert, Noens and Wagemans [120] used a gaze-contingent display paradigm to examine whether children with ASD show a preference for holistic face processing. This paradigm allowed participants to view the whole face (full), or the remaining part of the face outside the fixated region (mask), or solely the fixated region (window) when participants were presented with two faces and were instructed to decide whether the faces were the same or different. The behavioural results suggested slowed and less efficient face processing in ASD, compared to the TD group. When comparing the group difference in three different presentation conditions, results showed younger ASD children (6–10 years old) had reduced performance in the full viewing and mask conditions, compared to the TD group, but not in the window condition. This pattern was not observed in the older ASD group (10–14 years old). The eye-movement data representing visual exploration during the task, as indicated by heat map analysis, showed that younger ASD children had a dense heat map, in which a smaller area was centred around the fixation peak compared to the TD group, but older ASD children did not differ to TD controls. Therefore, younger ASD children scanned faces more narrowly compared to the TD group, and this finding fits with the previous findings discussed in the preceding text. Noteworthy, a further two studies from Yi and colleagues [121,122] explored viewing patterns for a face identification task, with an own race face and another race face condition, in Chinese children with and without autism. The findings from both the behavioural and eye-movement measures suggest a typical processing of own race face information, regardless of the atypicality observed in general face identification in both studies. Specifically, the two studies found that although the ASD group’s performance was reduced in the identification task compared to the TD group, the ASD group showed an expected advantage in identifying Chinese faces over Caucasian faces. Importantly, the eye movement analyses showed that all groups fixated for longer on the eyes but for shorter on the mouth in Caucasian faces compared to Chinese faces. These findings imply that any differences in face processing for faces from another race appear to be present in individuals with and without autism.

Further eye-movement studies designed to evaluate face processing differences in ASD have focused on multiple aspects of face information, including face identification, emotion recognition and the ability to infer mental states from faces. For example, Kirchner, Hatri, Heekeren and Dziobek [123] revealed that adults with autism spent less time fixating an emotional face presented in a naturalistic context and made more errors when they were asked to report the emotion of the face or to identify other characteristics from the faces (gender or age) compared to a TD control group. However, both groups showed longer fixation time on face and mouth regions in the emotion task relative to the identification task. Additionally, there was evidence of a positive correlation between fixation time on eyes and performance on an off-line face processing task (Reading the Mind in the Eyes Test, [124]) in the ASD group. This finding indicates that increased viewing of the eyes in a face is related to increased ability to infer the mental state of the face in the ASD group. Müller et al. [125] found the same relationship using dynamic social interaction stimuli and observed diminished pupil dilation only in ASD adults (pupil dilation can be used to infer an indication of interest or cognitive effort). The behavioural data from that study also showed the ASD group to have more difficulty to understand the mental states of the characters depicted in the interactions.

Some studies also support the view that longer fixation time on faces is related to emotion-processing skill in ASD [88]. For example, in an emotion-recognition task, Wieckowski and White [126] found a reduced ability to recognize disgust and sadness expressions in ASD, and this reduced ability was coupled with a finding of longer fixation times on the mouth in the ASD group compared to the TD group for these expressions. In further studies, autistic individuals have been shown to express emotion less appropriately in response to a person who was expressing emotions in a video, and they were also found to fixate less on the eyes of surprised faces. A comparison of fixation time between the correct and incorrect responses in both a recognition and expression condition revealed a more important role for the mouth than the eyes in modulating face-processing performance for specific expressions. A later study examined emotion recognition and face attention in ASD and schizophrenia across a broad range of contexts, where emotional faces were presented in isolation, digitally masked in emotional scenes, or were embedded in scenes with congruent or incongruent backgrounds [127]. Both clinical groups showed poorer recognition performance in all scene conditions, but this effect was absent for the isolated face condition. Compared to the masked scene condition, fixation time (%) to the face region was increased in unmasked scene conditions for all three groups. However, only the ASD group failed to show an increase of face viewing in the congruent scene condition relative to the incongruent scene condition, and this finding points to a subtle but specific atypicality of utilising face information in congruent contexts in ASD. 

Neutral faces appear to have special significance in ASD. For example, Tottenham et al. [78] revealed atypical processing of the eyes in neutral faces, and this was observed in behavioural performance, eye-movement measures, and brain activity. They found that participants with ASD spent less time on the eyes when viewing neutral faces compared to the TD group. They also showed an increased response in Amygdala activity which was larger in the neutral face condition compared to an angry face condition in ASD, and especially in an eye-gaze manipulation condition, where participants were cued to look at the eyes directly. Behavioural data from the isolated off-line threat rating and expression coding tasks suggested that the ASD group made more errors to label the expression of neutral faces, showing a tendency to perceive them as negative stimuli. Increased error rates were also related with a higher likelihood to evaluate neutral faces as the threatening stimuli in the ASD group relative to the TD counterparts. More importantly, more threatening scores in neutral face ratings in ASD indicated shorter fixation time on eye regions. These findings from the behavioural, eye-movement and brain-activity measures consistently indicate that neutral faces are processed differently in the ASD group.

One explanation for this atypicality could be that neutral faces are perceived as more ambiguous in social information for the ASD group, and hence, shifting fixation away from the eyes of neutral faces may be adopted by autistic individuals as a compensatory strategy to alleviate any potential threatening feeling associated with neutral faces. However, a very recent study from Wang, Lu, Zhang, Fang, Zou and Yi [128] found evidence of eye avoidance exclusively for angry faces in ASD children compared to TD controls. There have also been other inconsistent reports for eye avoidance. For example, Moriuchi, Klin, and Jones [129] found evidence to support eye indifference but not avoidance, whereas Kliemann, Dziobek, Hatri, Steimke, and Heekeren [130] found evidence to support both avoidance and indifference to eyes in ASD. Despite these inconsistencies, the atypical visual processing of faces may be a significant indicator of emotional processing differences in ASD.

### Key Findings

- Autistic individuals may show a reduced propensity to allocate attention to the eye region of faces, relative to TD individuals, which may reflect avoidance or indifference;
- Face processing may be less holistic in ASD with less allocation to core features and less saccades between these features, in comparison to TD individuals;
- Differences in the allocation of attention to facial features may be more pronounced for young children with ASD relative to older individuals;
- The use of face information to infer mental states results in longer processing times upon eye regions in autistic individuals relative to TD individuals;
- A reduced propensity to fixate the eyes has the potential to account for differences in emotion recognition in ASD.

## 15. Face-to-Face Interaction Paradigms

**Figure 6:**
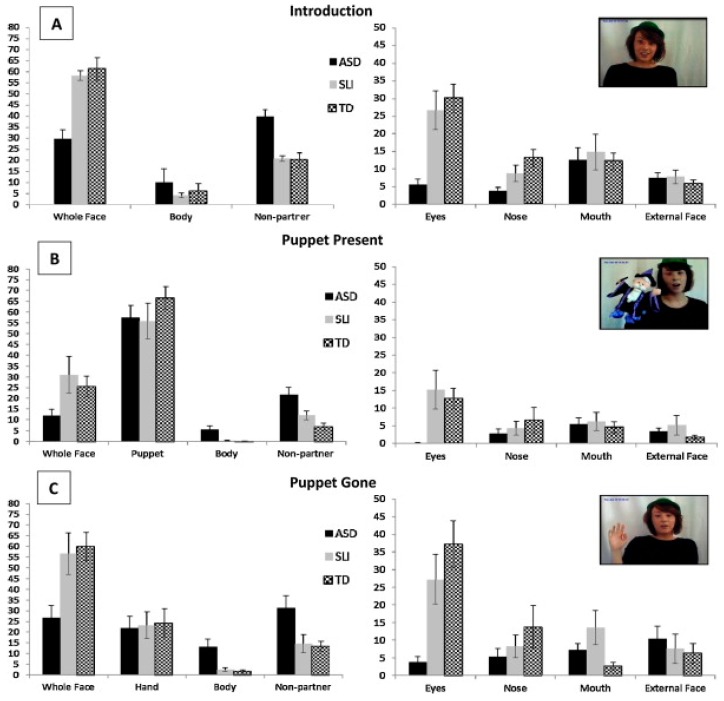
Percentage gaze to areas of interest for each section and for each group during a face-to-face interaction in Hanley et al.’s research [62]. Reproduced with permission from Elsevier.

Some studies have further extended the findings of atypical social attention observed in static or dynamic stimuli, to actual face to face interactions, where individuals with autism are required to engage with an interlocutor [62,131,132]. Riby et al. [132] investigated the gaze patterns when participants with autism and individuals with Williams syndrome (WS) were engaged in a question and answer interaction. A video recorder was set up behind the experimenter to monitor the eye gaze behaviour of the participants. Similar to the TD group, both the ASD and the WS participants showed more gaze-aversion behaviours (GA, total viewing time spent on non-questioner areas) when the question difficulty increased, and, both groups showed reduced performance when they were asked to look directly at the questioner throughout the whole session relative to free viewing. In addition, the ASD group made more GA compared to the TD group when listening to the interlocutor [131], and they also showed a weaker tendency to maintain their attention to the questioner during the whole interaction compared to the TD group [132]. Hanley et al. [62] presented further evidence of reduced attention to an interlocutor’s face, especially the eye regions, and this was coupled with increased attention to the non-face screen parts in an ASD group during a real live interaction in comparison to a TD group and to a group of participants with specific language impairment (SLI). A further subtle but significant finding showed that when an accidental event happened to a puppet in the interlocutor’s hand, ASD participants took significantly longer to begin to monitor the face of the interlocutor compared to both TD and SLI participants (see Figure 6). These findings are indicative of an ASD-specific difference in the allocation of social attention, and this is consistent with previous studies [98,111,128,131]. Delays in looking to the interlocutor’s face indicate that participants with ASD, unlike TD counterparts, did not immediately use the interlocutors face as a social reference marker to indicate detection of the ‘accident’.

### Key Findings

- Research adopting real-time face to face interactions between autistic and non-autistic individuals corroborates previous findings that task complexity may mediate social attention differences in ASD, and, that a requirement to share gaze with another throughout a task may be detrimental to performance.

## 16. Individual Differences

Eye-movement studies have also contributed to identifying individual differences in the field of social processing in autism for infants, children and adults, and have led to accounts as to how these could explain the inconsistent findings reported in relation to social processing in ASD. In particular, studies have shown that autism symptom severity, gender and comorbidity with other disorders might be a potential factor contributing to the mixed reports of differences in social attention or processing in autism [65,81,125,133,134]. For example, Bird et al. [65] found that for participants diagnosed with ASD, reduced preference to look at faces over non-face regions, presented in dynamic social interaction videos, was related to greater autism symptom severity, while lower preference scores for eyes over mouths was associated with alexithymia (defined as problems in recognizing and describing emotions). This finding highlights how ASD symptom severity may modulate face-processing atypicalities and that research exploring emotional processing in autism should consider the role of alexithymia, in addition to ASD [135]. Social anxiety and ASD are highly co-morbid [136] and both are related to atypical social attention. Kleberg et al. [81] has shown that autistic traits are associated with longer eye movement latencies to fixate another’s eyes, while social anxiety is associated with a greater tendency to avoid the eyes. Müller et al. [125] showed that there were two subgroups in their sample of ASD participants, with one group viewing less on the eyes and the other retaining similar fixation duration on the eyes as the TD group. These studies provide important evidence to promote consideration of the potential influence of autism symptom severity and other comorbidity on social processing in ASD.

Chawarska et al. [133] found that female infants at high risk for autism, between 6 and 12 months, showed increased attention to social scenes and to an interactive partner’s face compared to male high-risk counterparts and to low-risk female infants. In addition, the increased social attention in the high-risk female infants was related prospectively with less severe social difficulties at 2 years of age. However, this characteristic in female autism, observed in the infant stage, may disappear as development progresses over time. For example, Ketelaars et al. [134] has reported that adult females with autism looked for significantly shorter durations on dynamic faces showing intense emotions, as well as on the inner facial features, compared to TD adult females. This study also found a positive correlation between the time to first fixate on faces and social impairments in females with ASD relative to males, which is consistent with the findings of increased social attention (e.g., to face, eyes) and appropriate social behaviours reported from studies that tested male-dominant ASD groups [66,68,70,71,73,89,99,107]. What the studies cited above suggest is that social communication in autism may develop differently in female and male infants, and that social competence is linked to early orientation and sustained attention for social stimuli. However, it is noteworthy to point out that regardless of gender; there consistently exists a relationship between symptom severity and social processing proficiency across a wide range of ages in ASD.

In terms of the relationship between looking at the mouths of dynamic faces and abilities of social communication, results have been mixed. Johnel et al. [82] found that, when watching dynamic films with a speaking person, young children with autism who showed longer viewing time on the mouth tended to have more severe symptoms of ASD, whereas, Ketelaars et al. [134] revealed a reverse pattern in female adults with autism, who showed that those with more severe symptoms of ASD fixated less on the mouth. Elsabbagh et al. [83] have suggested that the relationship between mouth viewing in 7 months and expressive language (EL) at 36 months was modulated by the complexity of social stimuli for all the toddler participants. Specifically, endogenous control of attention to the mouth in a complex condition is associated with superior EL, and increased exogenous attention, driven by the repetitive mouth moving, is related to poorer EL. However, Rice et al. [75] found that this relationship varied significantly based on intelligence quotient (IQ) score characteristics. ASD individuals with higher verbal IQ relative to non-verbal IQ looked longer on mouths and also showed fewer social impairments, while ASD individuals who had higher full-scale IQ scores and no differences between verbal and non-verbal IQ, showed a reverse pattern. What the findings from the individual difference studies indicate is that within the ASD groups, autism symptom severity is not the only factor to impact upon processing of social information. Furthermore, increasing attention to the social world may be adaptative in ASD to aid communication in social interactions.

### Key Findings

- Social processing differences observed in autistic individuals may also be related to co-morbid conditions, such as social anxiety disorder and alexithymia;
- Based upon eye-tracking data, social processing differences may be more prominent for males with ASD in early life, relative to females;
- Symptom severity is associated with less adaptive social processing.

## 17. Social-Processing Summary

In summary, many studies employing eye tracking technology over the past 10 years have revealed subtle but significant differences in social attention in ASD. There is clear evidence for differences in the way autistic individuals allocate attention to social information and this may influence their response to such information. These differences predominantly appear to reflect either an *absence* of attention towards social information or *reduced propensity* or *delay* to sample this information. This atypicality in ASD could influence the seamless nature of interactions, or the detection of social cues in everyday exchanges. Ongoing events in everyday communication happen very rapidly, and, some social cues are implicit or ambiguous. Therefore, the subtle differences revealed by the eye-movement patterns in the studies reported in the social processing sections of this review, may be amplified in real social situations in ASD. This has clear potential to result in difficulties in understanding, following, keeping track of, and preparing and executing appropriate responses during ongoing communication.

## 18. Overall Conclusions

This review summarises the research that has used eye tracking over the past decade to examine cognitive processing differences in ASD, with a focus on social and language processing. The aim has been to identify how observed differences in processing could relate to behavioural symptoms that manifest in day to day communication.

In relation to the previous review into eye movements in ASD [5], issues that were raised from that review, such as the modulating effects of symptom severity, developmental stage, gender, co-morbidity, stimulus and social complexity, and, task demands on performance, are just as relevant for the studies presented in the current review, and will continue to be factors that need to be addressed in research into cognitive processing. A further factor when conducting such research relates to difficulties in recruitment of sufficient participants to carry out individual difference analyses. It is important that researchers work (possibly in a more collaborative way) to overcome such challenges to promote in-depth investigation into how these factors and diagnostic status influence on-line social and language processing, at an individual level, in the heterogenous autism population. Although there are many apparent inconsistencies in the research discussed in the current review, there are also some consistent eye-movement patterns, from both language and social processing studies that indicate how both temporal and spatial processing differences in autism might impact upon everyday communication.

From the language-processing literature, it would seem that there are subtle differences in how children with autism acquire language, and these differences may in part relate to how children with autism attend to and learn from the social environment. For example, in younger children some eye-movement studies have revealed that a reduced propensity to utilise an interlocutor’s gaze to support the mapping of referents upon new words. For older children and adults, few differences in language processing are reported when paradigms have low pragmatic and social response demands (e.g., visual world, reading). In these instances, differences are predominantly reported for higher-level language processing such as inferential work and in those studies the eye-movement patterns reflect quantitative delays in the time-course of processing relative to comparison groups.

Future research would do well to focus upon identifying more specifically when and why differences in the time course of language processing occur in ASD and how this interacts with social and sensory processing. For example, it is important to consider the language demands of instructions for social tasks when considering these findings. Previously, social, language, and sensory processing have predominantly been examined independently, yet they are inextricably linked.

From the social-processing literature, one predominant finding is that the saliency of social information may not be prioritised in infants, toddlers, children, adolescents and adults with autism. For example, social information may not be initially fixated by autistic individuals, the time taken to fixate important social information is greater, social cues may not be followed in the same way, core facial features may not be fixated as frequently, and there may be a reduction in eye movements between core facial features. These differences in early attention to social information highlight the importance of analysing the time-course of attention allocation during experimental trials.

Since the previous review, much more research using dynamic stimuli to examine the influence of task and stimulus complexity has been conducted. For example, the nature of social information presented in the task influences processing efficiency (e.g., one person versus multiple people in dynamic videos, and physical versus social oddities in static scenes). However, there now needs to be a focus upon specifying more discretely how tasks and stimuli may increase in complexity and how such increases will influence on-line processing for both TD and autistic individuals. This may be achieved by systematically manipulating the information that is made available, by analysing whole scan paths for each trial rather than single regions of interest, and by adopting a range of different eye-movement measures that can inform as to whether any group differences reflect differences in early detection or processing, or differences in later processing.

The subtle differences in language and social processing reported in the current review have the power to influence the nature of communication and interaction in ASD. For example, a reduction in the use of eye contact and differences in the speed of detection of (often implicit) communication cues in both language and visual social stimuli have clear consequences for everyday interaction. Moreover, differences in the use of such information in children with ASD may impede the development and exposure to various social schemas and contexts, which may affect social development per se.

One fairly consistent eye-movement pattern in adults with autism clearly shows that there may be a reduction in the speed with which attention is initially oriented towards social information, and this finding is especially relevant when viewing complex social scenes, or when engaged in complex dynamic tasks. Furthermore, from the scene perception studies we know that when odd social events are depicted, there is a failure to detect this information upon first fixation in ASD. Similarly, in the language-processing research, any differences that are observed tend to reflect a delay in the detection of target words or phrases that have some higher-level linguistic manipulation e.g., some types of inferences and implausibilities. This finding highlights that processing differences for complex tasks and/or stimuli may extend across different processing domains in ASD and that the type of processing differences observed, when examined on a moment-by-moment level, may be similar in nature. A second important eye-movement pattern shows that adults with ASD repeatedly fixate information in scenes, and they repeatedly re-read text (after reading this through once in entirety). This pattern is absent in the TD population. It is not clear why this pattern occurs in ASD, but it may be necessary for a coherent representation of a scene or text passage to be developed. For many studies this detailed analysis of the eye movement data is not performed, which means that it is not known whether repeated sampling occurs as a matter of course in ASD, when there is unlimited time to complete the task at hand. Hence the emphasis here is to illustrate the importance of the quality of the temporal and spatial eye-movement data, and the very detailed and sophisticated analyses of the eye-movement data, if we are to improve our understanding of how differences in on-line cognitive processing might contribute to difficulties in communication in the real world for individuals with autism. If repeated sampling is informative and can support communication for autistic individuals, the lack of opportunity to engage in repeated scanning of information in everyday communication will impede interaction, since day-to-day communication is typically fast and dynamic.

In conclusion, eye-movement studies have been instrumental in increasing our understanding of the drivers that might underpin manifestations of communication differences in ASD. The findings from the language and social-processing fields align to a degree and highlight that some processing differences are similar in both of those domains. Consistent reports of a failure to immediately orient to, detect or comprehend significant social cues, coupled with a propensity to resample information in scenes and reading, means that information that may be important to ongoing processing in the real world, which may be ‘missed’, cannot be sampled again, and this will have important consequences for keeping track of events, and for preparing and executing appropriate responses in everyday communication.
